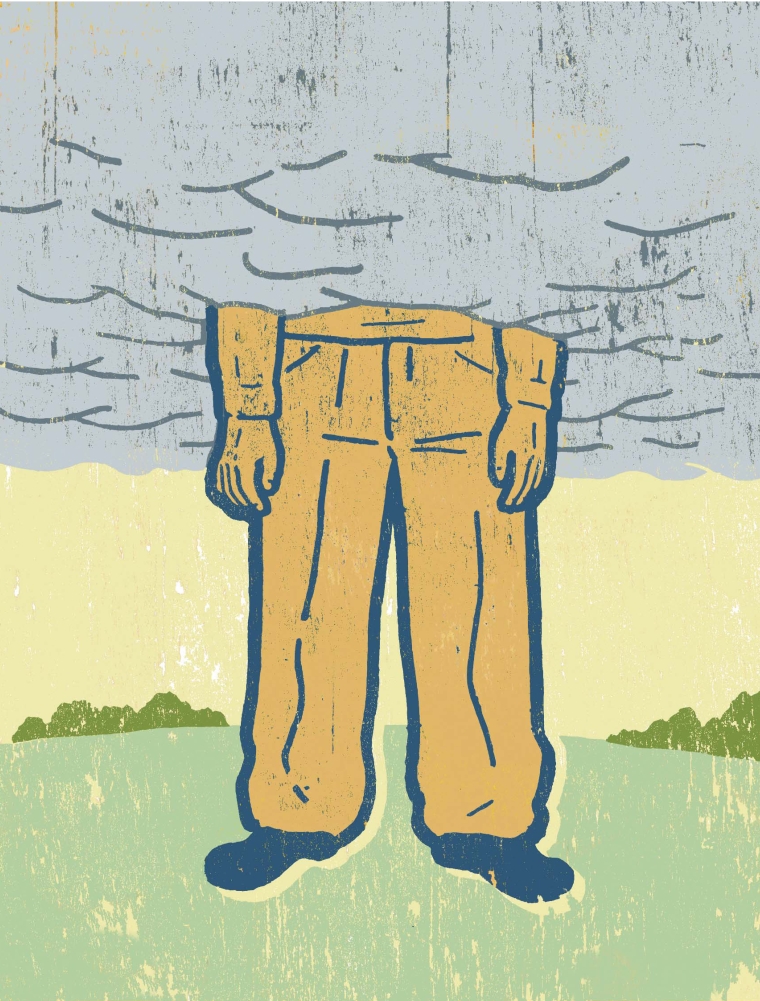# Ozone Nation: EPA Standard Panned by the People

**DOI:** 10.1289/ehp.116-a302

**Published:** 2008-07

**Authors:** Bob Weinhold

Under the Clean Air Act, the U.S. Environmental Protection Agency (EPA) is required to review the science underpinning the National Ambient Air Quality Standards to ensure those standards are adequately protective of public and environmental health. But in the decade-long deliberations over the EPA’s update of the ground-level ozone standard, science and regulatory action had a head-on collision.

A great deal of the science on the health effects of ozone suggests that the primary (i.e., public health) standard of 80 ppb that had been in place since 1997 was far too high to adequately protect the health of many U.S. residents. The EPA’s own scientists as well as its Clean Air Scientific Advisory Committee (CASAC)—the statutorily established body that advises the EPA administrator on the National Ambient Air Quality Standards—recommended going as low as 60 ppb to provide adequate protection in accordance with the mandates of the Clean Air Act. The World Health Organization (WHO) recommends an even lower level of 51 ppb.

But meeting such levels promises to be difficult and costly. Only a few counties in states such as Alaska, North Dakota, South Dakota, Montana, Washington, Oregon, and northern California would be likely to meet a standard of 60 ppb. Not that many more would be likely to get a green light at 65 ppb. Possibly the only place in the United States that meets the WHO guideline of 51 ppb is Hawaii.

That left EPA administrator Stephen Johnson in a tough position: weighing the science of more than 1,700 new studies reviewed by the agency against the possible fallout if he declared a vast proportion of the country out of compliance. However, the Clean Air Act specifically states that neither CASAC nor the administrator can take into account the cost or the ability to implement new standards in making their recommendations or in setting standards, says CASAC chair Rogene Henderson, scientist emeritus at the Lovelace Respiratory Research Institute.

In the end, Johnson set the standard at 75 ppb. The decision, announced 12 March 2008, elicited howls of protest from all sides, including some in Congress. Henderson says numerous state and local government officials, air pollution control authorities, environmental groups, and public health and medical organizations—including the American Medical Association, the American Thoracic Society, the American Academy of Pediatrics, and the American College of Chest Physicians—all vigorously supported lowering the ozone standard, and even supported a lower standard than was eventually set.

At the same time, scores of industry organizations and other state and local government officials chastised the EPA for its new standard, saying it shouldn’t have been tightened at all. National Association of Manufacturers president John Engler said in a 12 March 2008 press release that “(t)he costs are too high and the benefits too unclear to impose this new burden on America’s manufacturers and employees.”

Legal or congressional action launched in response to the decision could yet alter Johnson’s decision. In the meantime, Washington, DC, and about 350 counties—and possibly hundreds more—may need to soon begin taking steps to comply with the 75-ppb standard, according to the EPA. Even at this upper end of what scientists consider an acceptable range, half or more of the population could now be living in counties that have officially unhealthy levels of ozone.

## Complex Chemistry

Ground-level ozone is formed as a by-product of atmospheric reactions between nitrogen oxides and volatile organic compounds, precursor chemicals that typically come from sources such as factories, power plants, vehicles, and consumer products, as well as from natural sources such as vegetation. Ozone forms in the presence of sunlight, but the complex atmospheric chemistry of ozone formation is also affected by weather, land-forms, altitude, and many other factors, making it difficult to predict ozone concentrations based solely on the locations of the emission sources. “Ozone forms in places far from where the pollution is actually emitted,” says Paula Davidson, the U.S. National Weather Service’s manager for air quality forecast capability. [Ozone forecasts are available at http://www.weather.gov/aq/.]

The EPA uses ozone as an indicator of ozone itself and many other potentially toxic intermingled pollutants. Some of the primary health effects that are linked with ozone and its associated pollutants include premature death, upper and lower respiratory diseases such as asthma and bronchitis, heart attack, and other cardiovascular problems. EPA staff acknowledge that ozone levels as low as 40 ppb can cause health effects in susceptible populations. Several independent studies, including work by Michelle L. Bell and colleagues published in the April 2006 issue of *EHP*, suggest that premature mortality may occur at background concentrations as low as 10–25 ppb.

**Table:** Comparison of Proposed and Actual Ozone Standards Against Former Standard of 80 ppb

	Number of Cases Averted by 2020	
End Point	at 75 ppb	at 65 ppb
Premature death	260–2,300	940–7,100
Heart attack	890	2,300
Upper respiratory symptoms	4,900	13,000
Lower respiratory symptoms	6,700	17,000
Chronic bronchitis	380	970
Acute bronchitis	1,000	2,600
Asthma exacerbation	6,100	16,000
Lost work days	43,000	110,000
Lost school days	200,000	1,100,000
Hospital/emergency room visits	1,900	9,400
Minor restricted-activity days	750,000	3,500,000

**Note:** Nitrogen oxide controls needed for ozone reductions will also result in some reductions in particulate matter, included as “co-benefits” in the figures above.

**Adapted from:** U.S. EPA. 2008. Final ozone NAAQS regulatory impact analysis. Executive summary. Research Triangle Park, NC: Office of Air and Radiation, U.S. Environmental Protection Agency; Table ES.5.

The federal hammer used to encourage compliance with the standard is a threatened loss of federal funds by local or state recipients. However, over the past 30-plus years sanctions have been imposed in just 44 cases, says EPA spokeswoman Cathy Milbourn. Janice Nolen, the American Lung Association’s assistant vice president for national policy and advocacy, notes, “Federal transportation dollars are only withheld if the area fails to try to meet the standards.” Instead, that federal leverage tends to shift how the dollars are spent, says William Kovacs, vice president for environment, technology, and regulatory affairs for the U.S. Chamber of Commerce: “[Receiving parties] get the dollars, but they have to do different things with them, like bike paths or transit.”

## Teetering on the Edge of Compliance

The ozone standard is based on the three-year average of the fourth highest reading over an eight-hour period. That approach ignores the three highest readings and discounts high individual years, unlike guidelines from the WHO and the California Air Resources Board (set at 70 ppb), which allow no exceedances.

It’s uncertain which counties will exceed the EPA standard when the agency is scheduled to make its final determinations in 2010 or 2011. For instance, 85 counties with ozone monitors documented exceedance of the 80-ppb standard in early 2008, but that translated to 337 counties after the EPA and state and local officials estimated and negotiated which areas were in violation, accounting for those areas without monitors. For the new standard, the EPA says 345 counties with monitors exceed it now, and that could later translate to several hundred additional counties deemed to be in violation.

In any given county, it can matter a great deal which three-year period is picked (data from either 2006–2008 or 2007–2009 are the choices currently on the table) because the readings can vary by 15% or more from year to year. Monitor location also matters. Larimer County and Fort Collins, Colorado, looked like they were just sneaking in under the new standard, based on a central city monitor. But a new monitor added just four miles away in 2006 has registered readings averaging 86 ppb for 2006–2007. However, the lack of three full years of data at this particular monitor means that Larimer County isn’t officially out of compliance, although the EPA could readily determine through its estimation and negotiation process that Larimer County residents are contributing to local regional ozone problems and must take actions to reduce ozone.

Even areas of the country in settings often considered pristine are proving to have problems with ozone. One example is Wyoming’s Sublette County, which is one of many nodes of the rapidly expanding natural gas drilling industry throughout much of the western United States. Sublette County recently installed monitors that have shown people to be exposed to about a dozen peak one-hour readings at or above 100 ppb since 2005. The Wyoming Department of Environmental Quality issued five air pollution advisories in early 2008. “This is something we’re just not familiar with—high ozone in the dead of winter,” says Keith Guille, a spokesman for the department. “But these levels aren’t going away.” The 2005–2007 average for the fourth highest eight-hour maximum is 72.7 ppb, and it could be higher for 2006–2008.

Other epicenters of gas drilling and coal bed methane extraction activity include Campbell County in northeast Wyoming (with a fourth-highest eight-hour average of 69.0 ppb), San Juan County in northwest New Mexico (79.0 ppb at a two-year-old monitor), Lea County in southeast New Mexico (71.0 ppb), Eddy County and Carlsbad Caverns National Park in southeast New Mexico (69.7 ppb), and Montezuma County and Mesa Verde National Park in southwest Colorado (73.3 ppb). Many other national parks across the nation have been in violation of the new standard in the past few years, including Acadia in Maine, Cape Cod in Massachusetts, Shenandoah in Virginia, Great Smoky Mountains in Tennessee and North Carolina, Rocky Mountain in Colorado, Saguaro in Arizona, and Death Valley, Sequoia-Kings Canyon, and Yosemite in California.

The emphasis of the ozone standard on short-term peaks, which the EPA says is supported by the current science, doesn’t address long-term exposures. Much of the inland western United States, which routinely has ozone concentrations near or above the WHO standard all year long—and in some settings even throughout the night in the middle of winter, a period typically considered the lowest for ozone—is deemed to be adequately protected because short-term peaks above the EPA standard are relatively infrequent. The pattern in most of the eastern half of the country is completely different: ozone levels are relatively low for the cooler months and quite high in the summer and its flanking months.

## Living with the Standard, or Not

The links between specific health problems and seasonal, diurnal, climatic, and geographic variations in ozone exposure were highlighted as research priorities in *Estimating Mortality Risk Reduction and Economic Benefits from Controlling Ozone Air Pollution*, an EPA-requested report released by the National Research Council (NRC) on 22 April 2008. Other important science gaps the NRC identified included the poorly understood effects of co-pollutants and variations in personal responses to ozone. Nolen says the specific mechanisms through which ozone causes harm also need more investigation.

Independent studies have shown that another less-researched facet, indoor ozone exposure, likely is important for human health. Such exposures have typically been downplayed; although most people spend about 90% of their time indoors, outdoor ozone that inevitably infiltrates buildings hasn’t been widely considered a significant problem, in part because peak readings—in the limited number of cases where they’re taken—often are below the short-term maximum that determines the EPA standard.

But a research team from Lawrence Berkeley National Laboratory found a strong link between outdoor ozone and a variety of health problems among building occupants, such as neurologic and upper and lower respiratory symptoms. The problems occurred even when outdoor ozone levels were relatively low, according to two research articles by Michael Apte, Ian Buchanan, and colleagues published in the April 2008 issue of *Indoor Air*. And an October 2006 *EHP* review article by Charles J. Weschler demonstrated that although indoor ozone concentrations are lower than those in outdoor air, the amount that people actually took up over a 24-hour period was higher indoors than outdoors. Moreover, human intake of pollutants with indoor sources—including the toxic products of ozone’s reactions with other common chemicals—is roughly three orders of magnitude higher than intake of pollutants released outdoors, according to research in the September 2000 issue of the *Journal of the Air and Waste Management Association* by Alvin C.K. Lai and colleagues.

Should there be no delays in implementing the EPA’s new standard, hundreds of counties will soon need to begin following the EPA’s process to determine compliance with the new standard and begin mitigation procedures by 2013 or 2014. Those efforts could last through 2020 and far beyond.

However, the U.S. House of Representatives Committee on Oversight and Government Reform and the Senate Committee on Environment and Public Works are both scrutinizing the EPA decision, including allegations that the White House put undue pressure on Johnson to weaken the secondary ozone standard, which is intended to protect vegetation and ecosystems (in contrast to the human health protection conferred by the primary standard). Setting a secondary standard different from the one approved by Johnson could also alter ozone concentrations to which people are exposed.

On the legal front, numerous advocacy groups and state and local governments on all sides of the issue initiated lawsuits in late May 2008, challenging the EPA’s new primary standard for being either too high or too low. That legal process—likely to occur with all suits consolidated—could take a year or more to settle. Meanwhile, the schedule for implementing the new standard will remain unchanged unless the U.S. Court of Appeals for the District of Columbia, where the lawsuits were initiated, determines otherwise.

If congressional action or court decisions force the EPA to revise the standard, that would likely please CASAC, which sent a sharply critical letter to Johnson on 7 April 2008. The letter, authored by Henderson and 24 colleagues, read in part: “[T]he members of the CASAC Ozone Review Panel do not endorse the new primary ozone standard as being sufficiently protective of public health. The CASAC . . . unanimously recommended decreasing the primary standard to within the range of [60–70 ppb]. It is the Committee’s consensus scientific opinion that your decision to set the primary ozone standard above this range fails to satisfy the explicit stipulations of the Clean Air Act that you ensure an adequate margin of safety for all individuals, including sensitive populations.” The authors go on to write, “We sincerely hope that, in light of these scientific judgments and the supporting scientific evidence, you or your successor will select a more health-protective primary ozone standard during the upcoming review cycle.”

That review cycle is scheduled to conclude in 2013. Meanwhile, well over half the country’s inhabitants now live in counties that don’t meet even the modestly tougher standard. That leaves Nolen very concerned: “It’s clear to us this is a serious risk.”

## Figures and Tables

**Figure:**